# Dynamic Impact Properties of Carbon-Fiber-Reinforced Phenolic Composites Containing Microfillers

**DOI:** 10.3390/polym15143038

**Published:** 2023-07-13

**Authors:** Ibraheem A. Abdulganiyu, Oluwasegun. E. Adesola, Ikechukwuka N. A. Oguocha, Akindele G. Odeshi

**Affiliations:** Department of Mechanical Engineering, University of Saskatchewan, Saskatoon, SK S7N 5A9, Canada; qvf897@mail.usask.ca (O.E.A.); iko340@campus.usask.ca (I.N.A.O.); ago145@mail.usask.ca (A.G.O.)

**Keywords:** particle-reinforced composites, mechanical properties, split-Hopkinson pressure bar (SHPB), impact behaviour, crystallinity, fracture

## Abstract

The addition of nano- and microfillers to carbon-fiber-reinforced polymers (CFRPs) to improve their static mechanical properties is attracting growing research interest because their introduction does not increase the weight of parts made from CFRPs. However, the current understanding of the high strain rate deformation behaviour of CFRPs containing nanofillers/microfillers is limited. The present study investigated the dynamic impact properties of carbon-fiber-reinforced phenolic composites (CFRPCs) modified with microfillers. The CFRPCs were fabricated using 2D woven carbon fibers, two phenolic resole resins (HRJ-15881 and SP-6877), and two microfillers (colloidal silica and silicon carbide (SiC)). The amount of microfillers incorporated into the CFRPCs varied from 0.0 wt.% to 2.0 wt.%. A split-Hopkinson pressure bar (SHPB), operated at momentums of 15 kg m/s and 28 kg m/s, was used to determine the impact properties of the composites. The evolution of damage in the impacted specimens was studied using optical stereomicroscope and scanning electron microscope. It was found that, at an impact momentum of 15 kg m/s, the impact properties of HRJ-15881-based CFRPCs increased with SiC addition up to 1.5 wt.%, while those of SP-6877-based composites increased only up to 0.5 wt.%. At 28 kg m/s, the impact properties of the composites increased up to 0.5 wt.% SiC addition for both SP-6877 and HRJ-15881 based composites. However, the addition of colloidal silica did not improve the dynamic impact properties of composites based on both phenolic resins at both impact momentums. The improvement in the impact properties of composites made with SiC microfiller can be attributed to improvement in crystallinity offered by the α-SiC type microfiller used in this study. No fracture was observed in specimens impacted at an impact momentum of 15 kg m/s. However, at 28 kg m/s, edge chip-off and cracks extending through the surface were observed at lower microfiller addition (≤1 wt.%), which became more pronounced at higher microfiller loading (≥1.5 wt.%).

## 1. Introduction

Carbon-fiber-reinforced phenolic composites (CFRPCs) are used widely as structural materials in aeronautical and space industries due to their excellent properties such as low density, low thermal expansion, high thermal stability, high strength and stiffness, and excellent resistance to creep, fatigue, and corrosion [1,2]. Moreover, they can be turned into carbon-carbon composites by converting the phenolic matrix into amorphous carbon through pyrolysis [3,4,5,6].

Phenolic resins have attractive properties which make them good matrix materials for fabricating carbon-fiber-reinforced composites. Compared to other polymers, phenolic resins have lower flammability and smoke emission, superior mechanical strength, higher hardness, better heat resistance, chemical resistance, and dimensional stability [7,8,9]. Phenolic resin material systems have been used for ballistic protective body armor since the 1960s. Phenolic resin blended with polyvinyl butyral (PVB) resin was one of the earliest matrix materials approved for ballistic protective body armor [10]. In the early 1960s, DeBell & Richardson Inc. (Enfield, CT, USA) developed a PVB-phenolic resin system initially for nylon helmet liners. Aircraft structures experience impact damage as a result of bird strikes, turbine blade failures, and inspiration of runaway debris into the engine, with velocities of 100–150 m/s and impact energies of over 100 J [11,12].

The structural performance of polymers and fiber-reinforced polymer composites can be enhanced through the addition of filler materials such as carbon nanotube (CNT), graphene, graphene oxide (GO), nanoclay, non-metallic oxides and carbides, and metallic oxides, carbides, borides, silicates, and silicides. The modification of matrices of CFRP composites with nano- and microfillers has been reported to lead to considerable improvement in their ablation resistance [13,14,15,16,17,18,19], interlaminar shear strength (ILSS) [20,21,22,23,24,25,26,27], interlaminar fracture toughness (ILFT) [22,28,29,30], wear and tribological properties [31,32,33], as well as flexural properties (strength and elastic modulus) [34,35]. Xu et al. [13] studied the ablation resistance and mechanism of CF-fabric-reinforced phenolic resin composite modified with particles of tantalum disilicide (TaSi_2_) and reported significant improvement in the anti-ablation property of TaSi_2_-modified composite in comparison with the unmodified composite. At 50 wt.% TaSi_2_ addition, the linear ablation rate and mass ablation rate reduced by 12% and 30%, respectively. In a different study, Wang et al. [14] found that the linear ablation rate of SiC particle-modified CF-reinforced phenolic resin composite decreased with increasing SiC content up to 5 wt.%, after which it started to increase. The effect of halloysite nanoclay addition on the mechanical and thermal properties of carbon/glass-fiber-reinforced epoxy resin composites was investigated by Nagaraja et al. [20]. They found that the interlaminar shear strength (ILSS) of nanoclay-modified composites was higher than that of the unmodified composites. At 3 wt.% halloysite nanoclay content, the ILSS of the composites improved by roughly 18% over that of unmodified composites. Lyu et al. [27] reported roughly 55% and 94% increases in ILSS and flexural strength, respectively, for CF/polyetheretherketone (PEEK) composites modified with multi-walled carbon nanotube (MWCNT) when compared to unmodified CF/PEEK composites. Kostagiannakopoulou et al. [29] studied the effect of adding graphene nanoplatelets with different geometrical characteristics on the interlaminar fracture toughness (ILFT) of CFRP composites. They observed that composites containing graphene nanoplatelets with high values of aspect ratio (AR) and specific surface area (SSA) showed higher Mode I and II ILFT than the unmodified composites.

A main challenge arising from the inclusion of micro- and nanofillers in polymer-based composites is that they agglomerate during dispersion in the matrix as a result of their large SSA and van der Waals forces [36,37,38]. The net effect of agglomeration is that it negatively influences the end properties of the resulting composites. Moreover, pristine carbon fiber is smooth, chemically inert, hydrophobic, and possesses low surface energy. As such, bonding between it and the polymer matrix occurs mostly through the weak van der Waals forces. Some of the methods employed in dispersing fillers in polymer matrices are vigorous mixing, shear mixing, mechanical mixing (e.g., ball-milling and 3-roll milling or calendaring), ultrasonication, magnetic stirring, surface coating of fillers, and chemical and physical surface functionalization [29,36,37,38,39,40,41]. While a perfect dispersion of the filler in different polymer matrices could be very challenging and difficult to achieve, factors such as the type of the polymer matrix (thermoplastic or thermoset), dimensions and amount of microfillers, the availability of techniques and fabrication processes are considered when selecting a proper technique for filler dispersion [29,41]. The dispersion state of fillers in matrices of polymer composites plays an important role in determining their overall performance. Its evaluation can provide an insight into the relationship between the microstructure and measured properties of the composites [42,43]. The experimental techniques commonly used for evaluating dispersion state include optical microscopy, dynamic light scattering (DLS), ultraviolet-visible-infrared (UV-Vis-IR) spectroscopy, scanning electron microscopy (SEM), transmission electron microscopy (TEM), X-ray diffraction, X-ray microtomography, and small angle scattering techniques, such small angle X-ray scattering (SAXS), small angle light scattering (SALS), and small angle neutron scattering (SANS) [42,43,44,45,46].

A survey of the available literature shows that, whereas several research works (Zhao et al. [47], Mohsin et al. [48], Rouf et al. [49], Lomakin et al. [50], Rouf et al. [51], Li et al. [52])have been carried out on the dynamic mechanical properties of plain CF-reinforced polymer composites, there is little information on the dynamic impact behaviour of microfiller-modified CFRPCs (Chihi et al. [53]) investigated using a split-Hopkinson pressure bar (SHPB) apparatus. Zhao et al. [47] used an electromagnetic SHPB to study the influence of strain rate and loading direction on the compressive failure behaviour of 2D triaxially braided CF fabric/epoxy resin composites at dynamic strain rates of 200 s^−1^ and 220 s^−1^. The results of the SHPB tests were compared with those of quasi-static tests performed at strain rates of 10^−4^ s^−1^ and 10^−2^ s^−1^. Both axial and transverse compressive strengths were found to increase with strain rate, which they attributed to change in the mechanisms of damage evolution. Rouf et al. [49] studied the effect of strain rate on the compressive failure characteristics of a 1D non-crimp fabric (1D-NCF) carbon fiber/epoxy composite at strain rates of 0.003/s, 0.2/s, and 325/s. The high strain rate (325/s) test was performed using a SHPB apparatus. A strong strain rate dependency of the compressive strength of the composites was reported. On the other hand, Li et al. [52] studied the strain rate dependency of mechanical properties of warp-knitted and plain weave CF fabric/epoxy resin composites. A quasi-static test was performed at 0.5/s, while dynamic compression tests were performed with a SHPB instrument at strain rates that ranged from roughly 221/s to 1337/s. A weak strain rate dependency of the dynamic compressive strength was reported. Chihi et al. [53] investigated the dynamic response and damage characteristics of CF/epoxy resin composites containing different weight fractions of CNTs (0%, 0.5%, and 2%) using the SHPB equipment. Compression SHPB tests were conducted at impact pressures ranging from 1.4 bars to 2 bars. The results showed that the addition of CNTs significantly improved the mechanical performance of CF/epoxy composites. Within the range of CNT content and impact pressure tested, the maximum compressive strength obtained increased with the impact pressure and weight fraction of CNT.

The goal of this research is to improve the impact response of carbon-fiber-reinforced phenolic composites with microfillers (colloidal silica and micron-sized silicon carbide) at varying amounts and establish a performance threshold limit of the CFRPCs with the microfiller addition. This would help usher in a new class of high-performing CFRP composites for use in high strain rate applications without suffering catastrophic failure in service. In the long run, this research is intended to develop numerical models that could predict the CFRP material performance at high strain rate loading conditions. To develop this numerical model, material data generated at high strain rates are necessary. For this purpose, the SHPB device was used in this work to obtain high strain rates.

## 2. Materials and Methodology

### 2.1. Materials

**Figure 1:**
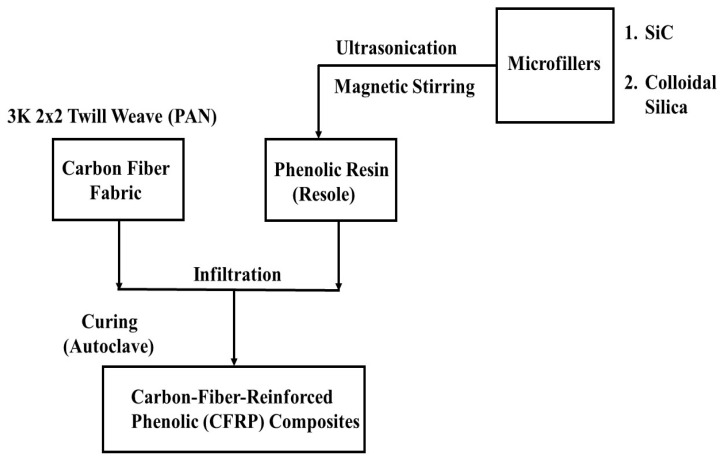
A flow chart showing the procedure for fabricating microfiller-modified CFRP composites.

Figure 1 shows the flow chart used in manufacturing CFRP composites containing microfillers. A 3K, 2 × 2 twill weave (PAN) carbon fiber (CF) fabric manufactured by Fibre Glast Developments Corporation (Brookville, OH, USA), with each fabric layer measuring 0.305 mm thick, was used as the carbon fiber material. Two resole-type phenolic resins (HRJ-15881 and SP-6877) procured from SI Group (Schenectady, NY, USA) were used as matrix materials. Their compositions and properties, as provided by the manufacturer, are shown in Table 1. Two microfillers were used to modify the matrices, namely: colloidal silica (406), which was procured from West System Inc., (Bay City, MI, USA), and silicon carbide (SiC) produced by Washington Mills, (North Grafton, MA, USA). The colloidal silica (CS) comprises sand and quartz particles with an average particle size of 0.2–0.3 μm and a density of 50 g/L. The density of the SiC was 3.19 g/cm^3^ and its particle size range was 0.3–2.2 μm. Polyethylene glycol (PEG) (product name: Carbowax^TM^ PEG-400), with a density of 1.13 g/mL, which was procured from Fisher Chemical Canada, was used to disperse the colloidal silica in the phenolic resins. PEG has long molecular chains which tend to disrupt the mutual attraction between individual microfiller particles, thereby promoting their uniform dispersion in the phenolic resins [37,38].

#### 2.1.1. Dispersion of Microfillers in the Resole Phenolic Resins

A detailed description of the dispersion of colloidal silica (CS) and SiC microfillers in the phenolic resins can be found in the work of Abdulganiyu et al. [54]. To provide further context, CS was mixed with PEG in a ratio of 2:1 to form a shear-thickening fluid (STF). By shear thickening, we mean an increase in viscosity with increasing rate of shear strain during mechanical deformation [55]. The PEG and CS mixture was stirred using a magnetic stirrer at 1200 rpm for 3 h at room temperature. Afterward, the resulting STF was diluted with ethyl alcohol using a ratio of 3:2 and held for 2 h. The mixture was then added to the phenolic resins in the appropriate amount to obtain 0.5, 1.0, 1.5, and 2.0 wt.% of CS in the resins. To promote CS dispersal in the phenolic resins, the mixture was stirred magnetically at 1200 rpm for 1 h, followed by ultrasonication for 1 h using a Branson 1510 ultrasonicator. The ultrasonication was performed in steps of 10 min with 5 min of rest in between to prevent overheating.

Firstly, SiC particles were weighed in the appropriate proportions to correspond to 0.5, 1.0, 1.5, and 2.0 wt.%. Then, they were mixed with 5 mL ethanol absolute to produce composites containing 0.5 wt.% SiC. The volume of ethanol mixed with SiC particles was increased to 6, 7, and 8 mL, for composites containing 1.0, 1.5, and 2.0 wt.% SiC particles, respectively. The main reason for using ethanol absolute was to help disperse SiC particles in the phenolic resin. In addition, it was anticipated that the ethanol would evaporate when the resole resin is heated during the curing process, leaving well-dispersed SiC particles in the phenol resin matrix of the resulting CFRP composite. The SiC particles-ethanol mixture was then ultrasonicated for a total of 30 min using the Branson 1510 ultrasonicator. The sonication was also carried out in time steps of 10 min with 5 min rest in between to prevent overheating. The ultrasonicated mixture of ethanol and SiC microfillers was then mixed with the phenolic resin and the new mixture was further sonicated for 1 h (in time steps of 10 min with 5 min rest in between to prevent overheating). Finally, after the ultrasonication, the mixture was magnetically stirred at 1200 rpm for 1 h.

#### 2.1.2. Fabrication of CFRP Composites

An in-house mold measuring 170 mm × 60 mm × 10 mm (*l × w × t*) was used to manufacture CFRP composite specimens used for dynamic impact testing. The carbon fiber fabric was cut into rectangular sheets measuring 160 mm × 60 mm (*l × w*). Since the thickness of the internal cavity of the mold is 10 mm and thickness of the carbon fiber fabric sheet is 0.305 mm, the number of reinforcement sheets used (*M*) was calculated using Equation (1).

(1) $$M=\frac{thickness\;of\;mold\;internal\;cavity}{thickness\;of\;carbon\;fiber\;fabric\;sheet}=\frac{10\;mm}{0.305\;mm}$$

The internal cavity of the mold was sprayed with a SLIDE^®^ Epoxease mold release agent (No. 40614N) produced by Infotrac (USA) and allowed to dry for 25 min. The lubricant made the removal the CFRPCs from the mold after fabrication easy. Using the hand lay-up technique, 33 sheets of the carbon fiber fabric were pressed together to fit into the 10 mm thickness of the internal cavity of the mold. Afterward, the previously prepared mixture of microfillers and phenolic resin was poured into the mold cavity and it slowly impregnated the carbon fiber fabric sheets with the help of gravity.

Curing of the resin-impregnated carbon fiber fabric was carried out in a Parr 4848 pressure reactor autoclave under an argon gas atmosphere at a pressure of 50 bar. The temperature of the autoclave was ramped up from room temperature to 120 °C at a heating rate of 2 °C/min and maintained at 120 °C for 1 h, after which it was cooled to room temperature. The argon gas atmosphere helped to remove gaseous by-products of polymerization reactions that occurred during curing. It also helped to reduce the porosity of the CFRPCs.

### 2.2. Characterization of Fabricated Materials

#### 2.2.1. X-ray Diffraction (XRD)

Specimens used for XRD experiment were prepared by dipping CFRP composites in liquid nitrogen and crushing them in a clean ceramic crucible to fine aggregates. XRD analysis was obtained with a Rigaku Ultima IV X-Ray Diffractometer (Rigaku Americas Corporation, The Woodlands, TX, USA) using Cu Kα source (*λ* = 1.54056 Å), tube voltage of 40 kV and tube current of 44 mA. Experimental data were collected using a Multipurpose Attachment, with para-focusing mode. A nickel K_β_ filter was placed at the receiving end. X-ray diffractograms were acquired in 2θ from 5° to 60° at a scan rate of 0.5°/min and a step size of 0.02. The software used for background correction and data smoothening was PANalytical X’Pert Highscore, while Origin 2019 (OriginLab, Northampton, MA, USA) software was used for plotting and analyzing the obtained XRD data.

#### 2.2.2. Dynamic Impact Test

**Figure 2:**
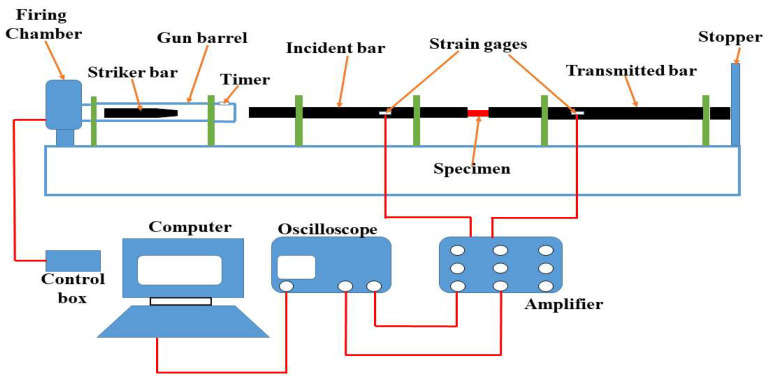
A schematic diagram of the split-Hopkinson pressure bar (SHPB) system.

**Figure 3:**
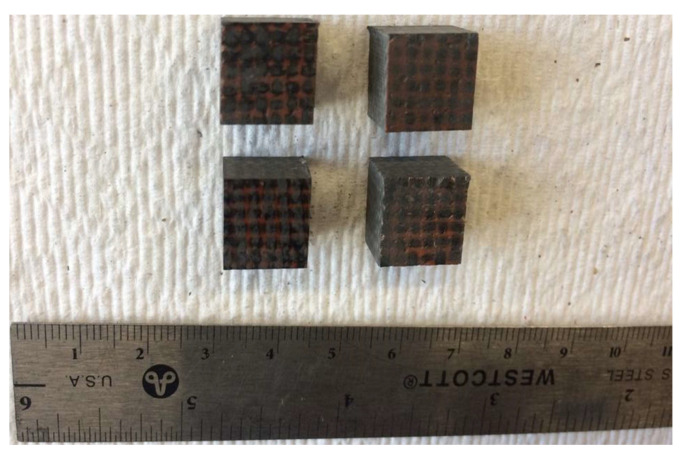
Photographic image showing typical dynamic compression test specimens.

The high strain rate behaviour of fabricated CFRP composites was investigated using a split-Hopkinson pressure bar (SHPB) apparatus, the schematic diagram of which is presented in Figure 2. As can be seen, it comprises three bars, namely: striker, incident, and transmitter bars. The transmitter and incident bars were machined from aluminum alloy 7075-T651 and have a diameter of 38 mm. Figure 3 shows a photographic image of typical impact test specimens, each measuring 12 mm × 12 mm × 10 mm. The surfaces of test specimens in contact with the incident and the transmitted bars were lubricated with Vaseline to reduce friction between the contacting end surfaces. With the specimen placed along the thickness between the incident and transmitted bars, the striker bar was fired with the aid of the gas gun toward the incident bar at pressures of 50 kPa and 80 kPa which corresponded to impact momentums of 15 kg m/s and 28 kg m/s, respectively. Five specimens of each CFRP composite and neat cured resin were tested at each impact momentum.

During SHPB tests, an incident stress pulse is produced when the striker bar impacts the incident bar which is propagated along the incident bar toward the specimen–incident bar interface. A portion of this stress pulse is back-reflected through the incident bar, while the remainder is propagated to the transmitter bar through the specimen. Since a constant strain rate helps to eliminate the radial inertia effects on test specimens during SHPB tests [56], a pulse shaper with a thickness of 12 mm but the same diameter as the incident and the transmitter bars was attached to the end of the incident bar. As such, on firing at a given impact momentum, the striker came into contact with the pulse shaper to help achieve a constant strain rate. Strain gauges were used to capture the generated elastic strain waves (incident, reflected, and transmitted) traveling through the incident and transmitter bars. Amplification and conditioning of the elastic strain waves were carried out using the connected strain conditioner-amplifier system. The output from the conditioner-amplifier system was linked to the mixed-signal digital oscilloscope which, in turn, was connected to the computer. The oscilloscope output was controlled and graphically displayed by in-house developed LabView software program. Based on stress equilibrium and obtained experimental data, the engineering or nominal stress ($\sigma_{e}$), engineering strain (${ɛ}_{e}$), and initial engineering strain rate $\left({\dot{\varepsilon }}_{e}\right)$ were calculated using Equations (2)–(4), respectively [57]:

(2) $$\sigma_{e}=\frac{A_{b}}{A_{s}}E_{b}{ɛ}_{T}$$

(3) $${ɛ}_{e}=-2\frac{C_{b}}{L_{s}}\int_{0}^{t}{ɛ}_{R}dt$$

(4) $${\dot{\varepsilon }}_{e}=-2\frac{C_{b}}{L_{s}}\varepsilon_{R}$$

where *A_S_* and *A_b_* stand for cross-sectional areas of the specimen and bars, respectively; ε*_T_* and ε*_R_* stand for transmitted and reflected strain waves, respectively; *C_b_* is the elastic wave velocity in the bars; *E_B_* stands for the Young’s modulus of the bar materials; while *L_s_* and t represent the original length of the specimen and deformation time, respectively. True stress (σ_t_) vs. true strain (ε_t_) curves were generated based on Equations (5) and (6), while the true strain rate $\left({\dot{\varepsilon }}_{t}\right)$ was calculated using Equation (7) [58].

(5) $$\sigma_{t}=\sigma_{e}(1-\varepsilon_{e})$$

(6) $$\varepsilon_{t}=-ln(1-\varepsilon_{e})$$

(7) $${\dot{\varepsilon }}_{t}=\frac{{\dot{\varepsilon }}_{e}}{\left(1-\varepsilon_{e}\right)}$$

The two firing pressures used in this study, 50 kPa and 80 kPa, generated average true strain rates of 880 s^−1^ and 2150 s^−1^ in the test specimens, respectively.

#### 2.2.3. Microstructure Examination

Specimens with dimensions 10 mm × 10 mm × 10 mm, cut from the fabricated rectangular plates of CFRP composites using a Buehler abrasive cutter (Model 95, C1800), were used for microstructure analysis. They were ground initially using 320, 500, 800, and 1200 SiC grit emery papers, followed by fine grinding using 2000 and 4000 SiC grit emery papers and finally polished using a 5 μm MD-Dac cloth with 5 μm MD-Dac suspension and a 1 μm MD-Nap cloth with 1 μm MD-Nap suspension. Microstructures of the polished and fractured specimens were analyzed using optical microscopy (OM, Nikon Optihot stereomicroscope, Melville, NY, USA, interfaced with a PAX-it digital camera) and scanning electron microscopy (SEM, JEOL-JSM-6010LV, Peabody, MA, USA). To reduce charging and improve the image quality, SEM specimens were gold-coated using an Edward S150B sputter coater (BOC Edwards, Crawley, UK). The SEM was operated at an accelerating voltage of 20 kV and images were acquired in the secondary electron (SE) imaging mode.

## 3. Results and Discussion

### 3.1. XRD

**Figure 4:**
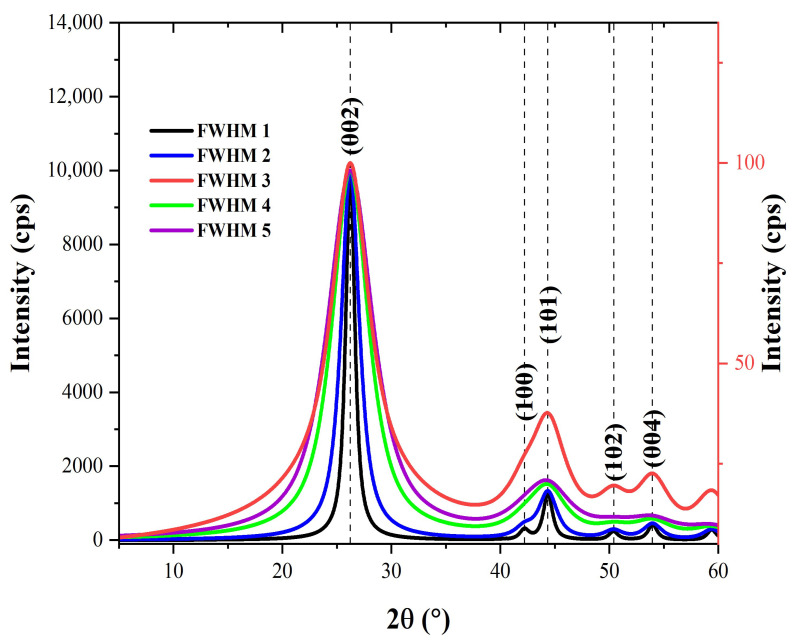
Diffraction patterns of graphite computed using different values of FWHM.

To properly index the X-ray diffraction (XRD) patterns obtained experimentally for microfillers and carbon fiber (CF), they were compared to standard diffraction peaks. Indexing of experimental diffraction peaks of the CF was carried out using the Mercury software (version 4.3.0) and graphite crystallographic information (.cif) file (deposition number: 918549). The graphite cif data were sourced from the crystal structure database of the Cambridge crystallographic data center (CCDC). The simulation of theoretical diffraction patterns of graphite was performed using full width at half-maximum (FWHM) values of 1.0, 2.0, 3.0, 4.0, and 5.0. The diffraction patterns generated using these FWHM values are shown in Figure 4. Clearly, the characteristic intense peak at roughly 2θ = 26.2° (002) is followed by minor peaks at 2θ~44.0° (101) and 2θ~54.0° (004). The existence of additional peaks at 2θ~42.3° (100) and 2θ~50.5° (102) depends on the FWHM value used.

**Figure 5:**
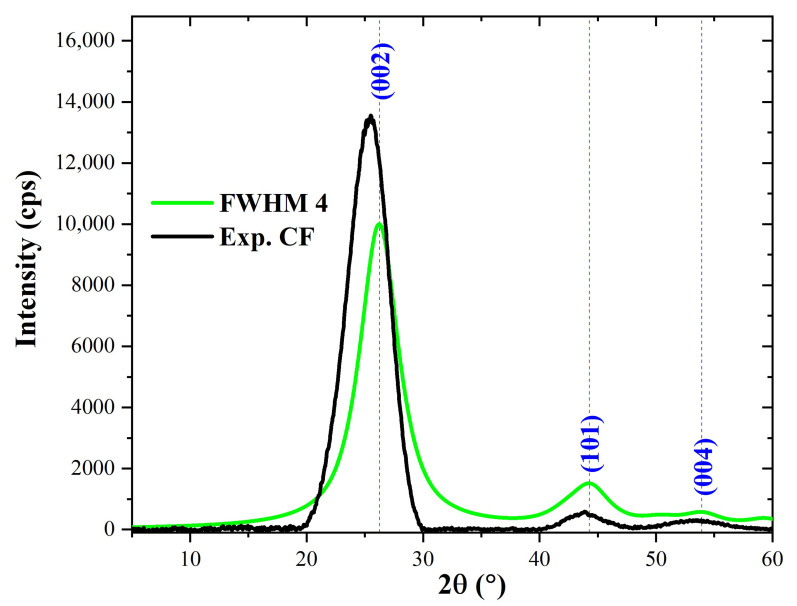
Diffraction pattern of graphite computed using an FWHM value of 4.0 compared with the experimental diffraction pattern obtained for carbon fiber (Exp. CF).

The calculated diffraction patterns were compared with the diffraction pattern obtained experimentally for the carbon fiber used in the present study (designated as Exp. CF). It was found that the XRD pattern calculated using an FWHM value of 4.0 matched the XRD pattern obtained experimentally for CF (Exp. CF). The two XRD patterns are co-plotted in Figure 5 for comparison. For Exp. CF, it can be seen that the characteristic peaks which appear at 2θ~25.7° (002), 2θ~44.1° (101), and 2θ~54° (004) are consistent with those obtained for the XRD pattern calculated using an FWHM value of 4.0. Nevertheless, a close look at the two diffraction patterns shows that the calculated (002) peak differs by 0.5° from that of Ex. CF, while the difference is 0.1° for the (101) peak.

**Figure 6:**
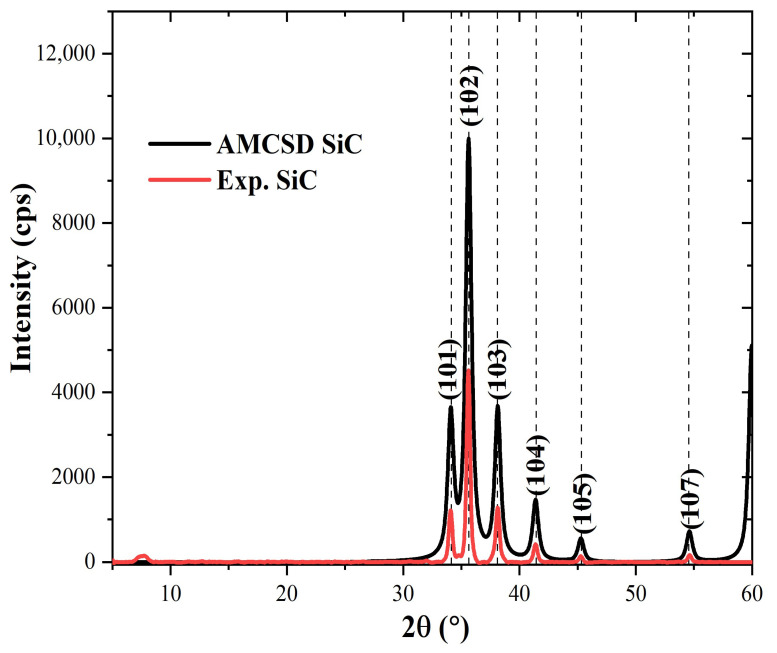
Calculated XRD pattern of α-SiC compared with that of the SiC microfiller (Exp. SiC).

SiC exists in different polymorphs with α-SiC (6H hexagonal) and β-SiC (3C-cubic) being the most stable structures [59,60]. While α-SiC possesses a hexagonal crystal structure and is made at temperature higher than 1700 °C, β-SiC has a cubic crystal structure and is prepared at temperatures lower than 1700 °C. Moissanite occurs naturally as SiC with the 6H hexagonal polymorph [61]. As such, it was used as a standard material with which to compare the SiC microfiller used in this study. The cif file of moissanite used in the present study was retrieved from the American mineralogy crystal structure database (AMCSD). The Mercury software was used to index the diffraction pattern of moissanite generated with an FWHM value of 0.6. The reason why 0.6 was used was because, as shown in Figure 6, the resulting diffraction pattern looked closest to that of the SiC microfiller (designated as Exp. SiC) used in modifying the matrix of CFRP composites developed in the present study. The characteristic features of the diffraction pattern of moissanite (α–SiC) (black colour) are diffraction peaks at 2θ~34.2° (101), 35.7° (102), 38.3° (103), 41.4° (104), 45.3° (105), 54.7° (107), and 60.9° (108). Several of these peaks appeared in the diffraction pattern of Exp. SiC (red colour), indicating that the SiC microfiller used is of the α–SiC type.

For colloidal silica (CS), its cif file could not be found to generate the calculated XRD pattern with which to compare the diffraction pattern of the colloidal silica used in this study (Exp. CS). However, for the Exp. CS, the indexing of the peaks was obtained and compared with values from the literature. Munasir et al. [62] and Jeon et al. [63] obtained (101) peaks for colloidal silica at 2θ~21.5° using the International Center for Diffraction Data (ICDD) PDF-2 database (PDF-2 No. 01-087-2096) and Inorganic Crystal Structure Database (ICSD) as standards. In the present study, a prominent peak at 2θ~21.76° was obtained for the colloidal silica corresponding to the (101) peak from the literature. We can infer that these values agree with one another, even though there is a 0.2° difference between the values reported in the literature and those obtained in this study. The corresponding XRD pattern of the colloidal silica microfiller is shown in corresponding XRD diffractograms, in relation to when colloidal silica microfiller is used.

**Figure 7:**
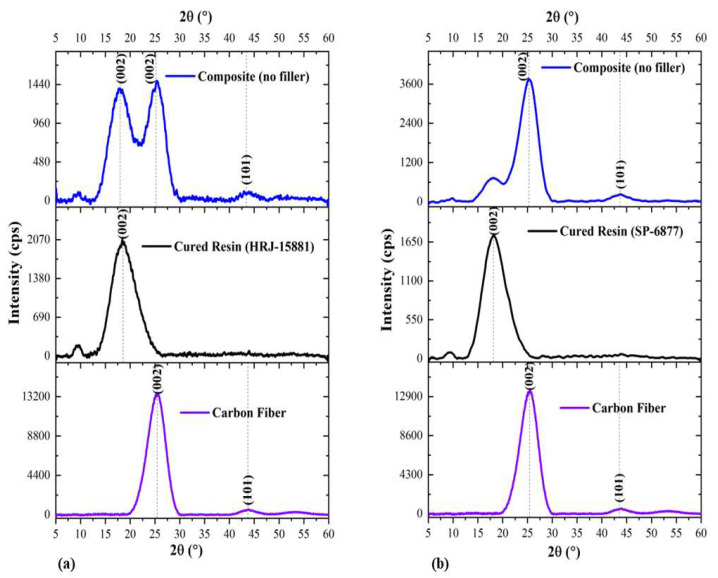
XRD patterns obtained for carbon fiber, monolithic resins, and their composites: (**a**) HRJ-15881 resin and its composite and (**b**) SP-6877 resin and its composite.

A comparison of XRD patterns obtained for CFRPCs made from phenolic resins HRJ-15881 and SP-6877 with those obtained for CF and monolithic resin is presented in Figure 7. Two conspicuous peaks at 2θ~18.8° and 25.7° for HRJ-15881 resin, and 2θ~18.6° and 25.7° for SP-6877 resin resulting from the addition of CF to the resins, can be seen. Obviously, this is due to the fact that the identities of the constituent materials were unaffected by the manufacturing process used in this study. Furthermore, it is seen that the intensity of the characteristic (002) peak of SP-6877 resin decreased, probably due to its low viscosity.

**Figure 8:**
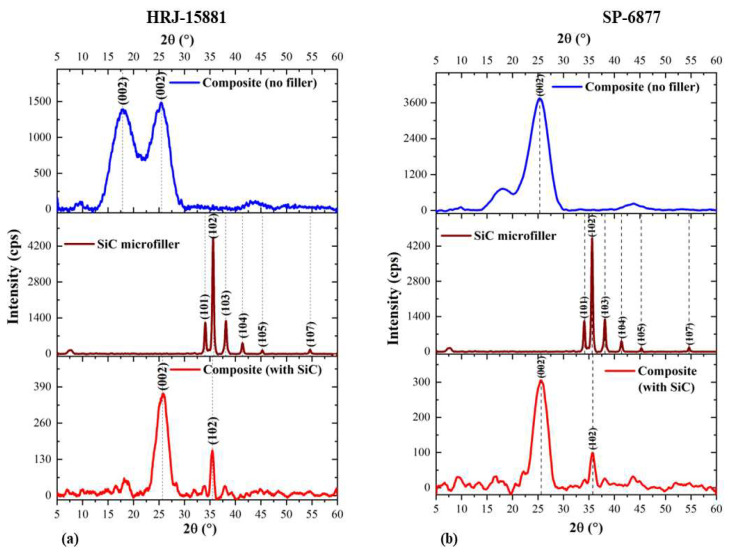
XRD diffraction patterns of CFRP composites modified with 0.5 wt.% SiC microfiller: (**a**) based on HRJ-15881 resin and (**b**) based on SP-6877 resin.

**Figure 9:**
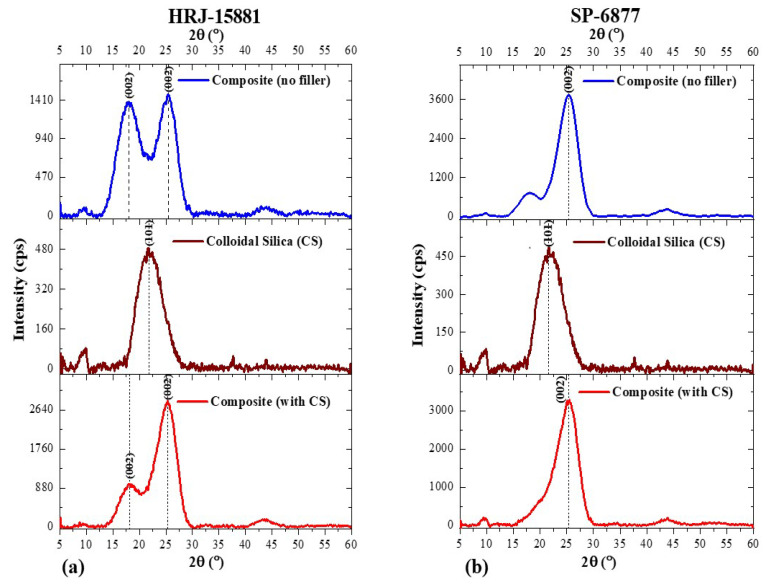
XRD patterns of CFRP composites modified with 0.5 wt.% CS: (**a**) HRJ-15881 resin and (**b**) SP-6877 resin.

Figure 8 and Figure 9 show, respectively, XRD patterns obtained for CFRPCs modified with SiC and CS microfillers for HRJ-15881 and SP-6877 resins. The addition of SiC filler to CFRP composites made with the two phenolic resins caused the intensity of the (002) peak to decrease. Sekhar and Varghese [64] investigated the thermal, mechanical, and rheological properties of phenolic resin modified with intercalated graphite bisulfate and found that the characteristic diffraction peak of the graphite moved to the left and its intensity reduced in comparison to the characteristic peak of natural graphite. Based on these results, it was concluded that the intercalated graphite truly intercalates and contains bisulfate. Also, Ki Park et al. [65] investigated the structural and electrochemical evolution of structured V_2_O_5_ microspheres during Li^+^ intercalation and found progressive changes in the characteristic diffraction peak of V_2_O_5_ as phase transformation occurred during Li^+^ intercalation. The characteristic (001) peak of V_2_O_5_ shifted left to a lower 2θ angle. The shift was attributed to α-V_2_O_5_ transformation to Li_x_V_2_O_5_ at the onset of Li^+^ intercalation. In the present study, the decrease in the intensity of the (002) peak of SiC-modified phenolic resin could be due to the penetration of the phenolic matrix by SiC microfillers. Furthermore, the characteristic (102) peak of α-SiC occurred at 35.7° for CFRPCs made with SiC-modified HRJ-15881 and SP-6877 phenolic resins.

Similar to the results presented in Figure 8, it is seen in Figure 9 that CS addition to the CFRP composites caused the intensity of the characteristic (002) peaks of HRJ-15881 and SP-6877 resins to reduce, which is an indication of the presence of CS in the respective matrices of the CF composites manufactured using both resins [64,65].

**Figure 10:**
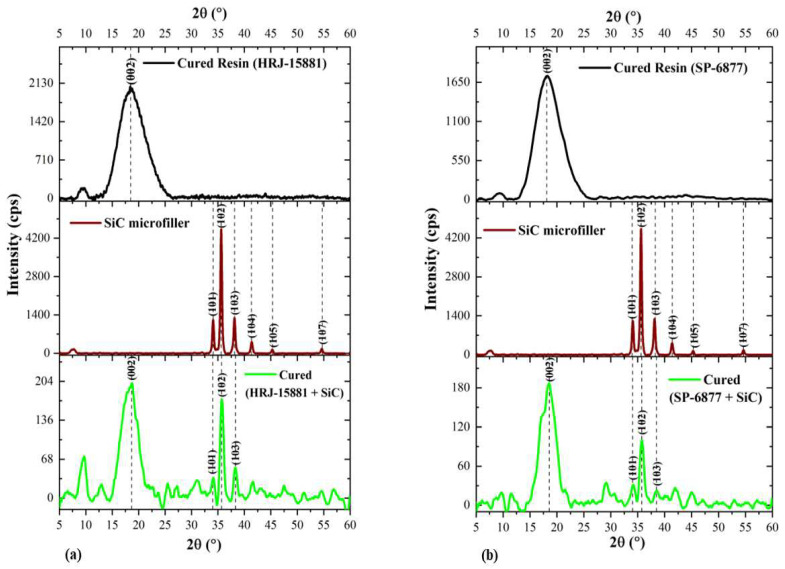
XRD patterns of phenolic resins modified with 1 wt.% SiC microfiller: (**a**) HRJ-15881 and (**b**) SP-6877.

**Figure 11:**
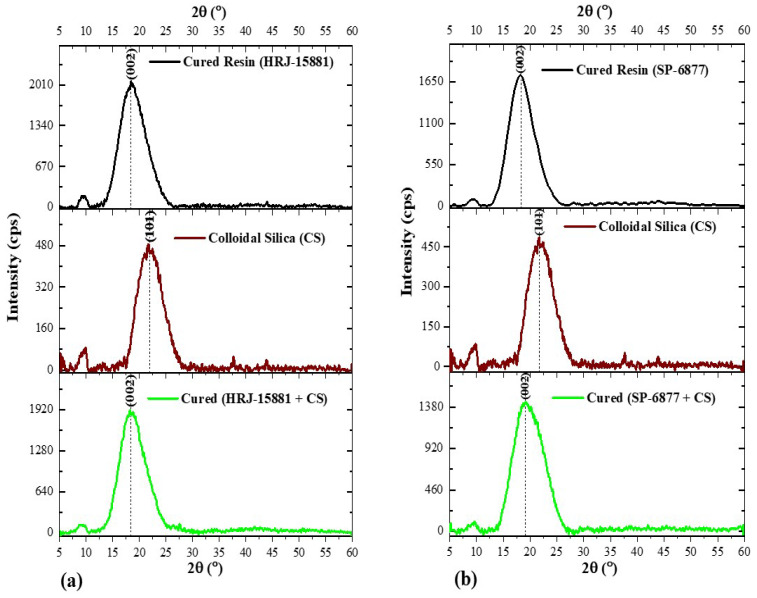
XRD diffraction patterns obtained for phenolic resins modified with 1 wt.% CS: (**a**) HRJ-15881 and (**b**) SP-6877.

Figure 10 and Figure 11, respectively, show changes in the intensity and angular position of XRD patterns of neat HRJ-15881 and SP-6877 resins due to the addition of SiC and CS microfillers. Figure 10 shows that SiC microfiller addition (1.0 wt.%) resulted in the existence of a (002) diffraction peak at 2θ~18.4° for both resins. The peaks at 2θ~34.2° (101), 35.7° (102), and 38.3° (103) are characteristic features of α–SiC as shown previously in Figure 8, which confirms that SiC microfillers are present and intercalated in the phenolics [64,65]. Figure 11 shows the effect of adding 1.0 wt.% CS to both resins. The prominent peak at 2θ~18.2° (green colour) shifted slightly to the left when compared with the characteristic peaks of individually cured resins which appeared at 2θ~18.8° and 2θ~18.6° for HRJ-15881 and SP-6877 resins, respectively. The peak shift and decrease in intensity due to CS addition may be due to its intercalation in the phenolic resins. Therefore, on the basis of the XRD patterns shown in Figure 11, it could be concluded that CS fillers are present and intercalated in both phenolic matrices [64,65].

### 3.2. Dynamic Impact Properties

#### 3.2.1. Impact Properties Obtained at a Momentum of 15 kg m/s

**Figure 12:**
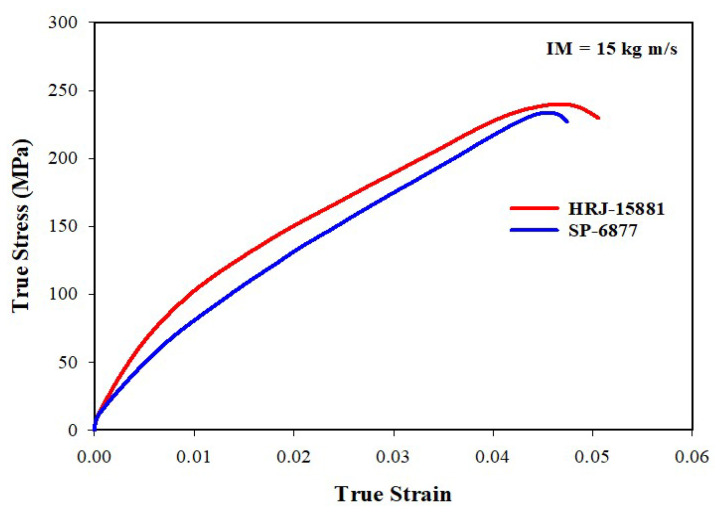
True stress–strain curves of unmodified CFRP composites (IM = 15 kg m/s).

**Figure 13:**
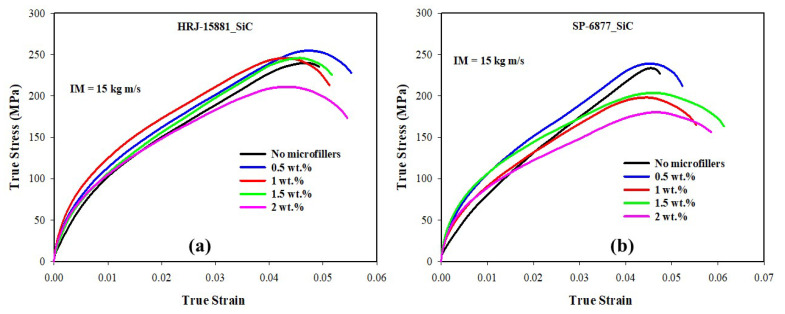
Typical true stress–true strain curves of SiC filler-modified CFRP composites (IM = 15 kg m/s): (**a**) HRJ-15881 and (**b**) SP-6877.

**Figure 14:**
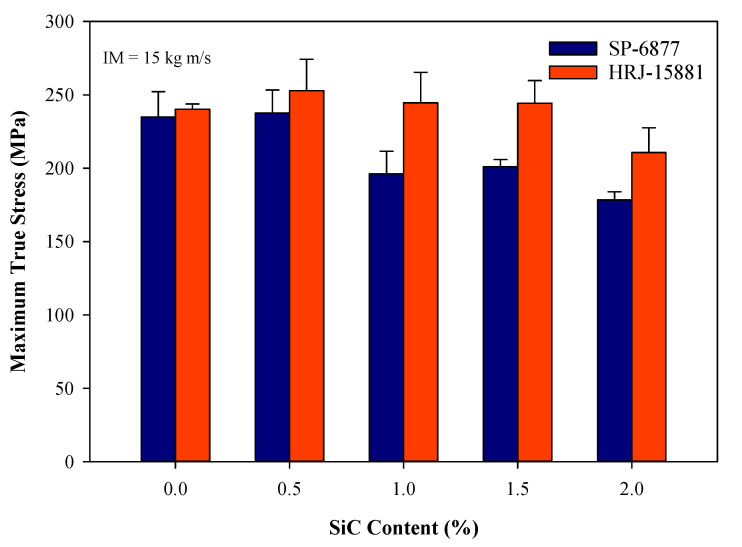
Comparison of maximum true stresses of SiC filler-modified CFRP composites made with the two phenolic matrices (IM = 15 kg m/s). Error bars are based on standard deviation.

**Figure 15:**
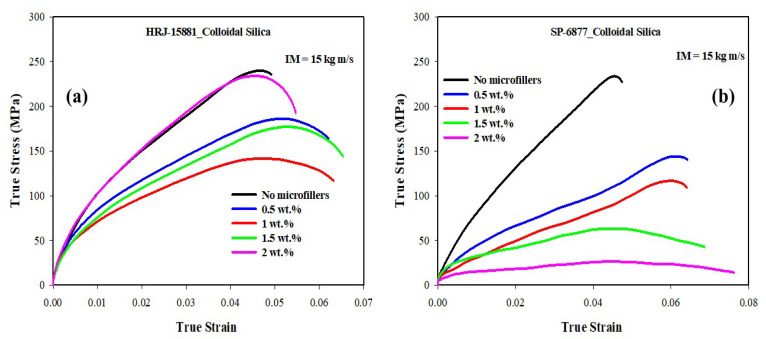
Typical true stress–true strain curves of CS filler-modified CFRP composites (IM = 15 kg m/s): (**a**) HRJ-15881 and (**b**) SP-6877.

**Figure 16:**
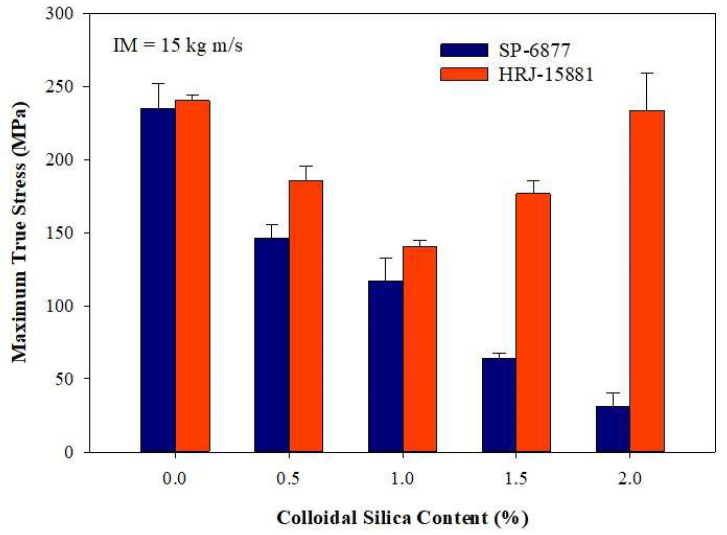
Comparison of maximum true stresses of CS filler-modified CFRP composites made with the two phenolic matrices (IM = 15 kg m/s). Error bars are based on standard deviation.

Figure 12, Figure 13, Figure 14, Figure 15 and Figure 16 present results obtained from SHPB tests carried out at an impact momentum (IM) of 15 kg m/s. Figure 12 shows the true stress–true strain curves obtained for unmodified CFRP composites. Figure 13 and Figure 14 show, respectively, the true stress-true strain and maximum true stress plots obtained for SiC-modified CFRP composites. Similarly, Figure 15 and Figure 16 present the true stress–true strain and maximum true stress plots obtained for CS-modified CFRP composites, respectively.

As shown in Figure 12, impact strength and toughness of the unmodified CFRP composites fabricated with HRJ-15881 phenolic resin are better than those of unmodified composites made with SP-6877 resin. A close examination of Figure 13 shows that up to 1.5 wt.% SiC addition, the CFRP composites based on HRJ-15881 matrix showed better impact strength than the unmodified CFRP composites. However, the impact strength of composites fabricated with SP-6877 resin improved over those of unmodified composites with 0.5 wt.% SiC addition, beyond which it decreased with increasing SiC content.

A plot of maximum true stresses obtained for CFRP composites modified with SiC microfiller is shown in Figure 14. The observed trend is similar to that of the true stress–true strain curves of Figure 13. It can be seen that the impact strength of CFRP composites based on HRJ-15881 matrix is higher than the impact strengths of those made with unmodified and SiC filler-modified SP-6877 phenolic matrix.

Figure 15 shows that the impact strength of CFRP composites modified with CS filler generally deteriorated with increasing microfiller content except for composites based on HRJ-15881 matrix with 1.5 wt.% and 2.0 wt.% CS. The cause of this discrepancy in impact strength within this range of CS content is not clear. The observed trend of reduction in impact strength with increasing CS content could be attributed to particle agglomeration. Abdulganiyu et al. [54] observed a deterioration in flexural properties with colloidal silica addition to CFRP composites owing to the agglomeration of colloidal silica particles. Nevertheless, a close look at Figure 16 indicates that the impact strength of CFRP composites based on HRJ-15881 matrix is higher than those based on SP-6877 matrix at all CS filler contents.

#### 3.2.2. Impact Properties Obtained at a Momentum of 28 kg m/s

**Figure 17:**
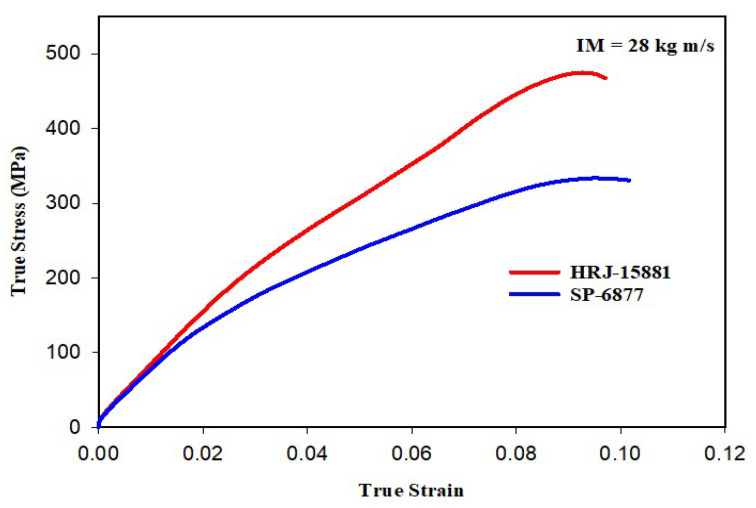
Typical true stress–true strain curves of unmodified CFRP composites (IM = 28 kg m/s).

**Figure 18:**
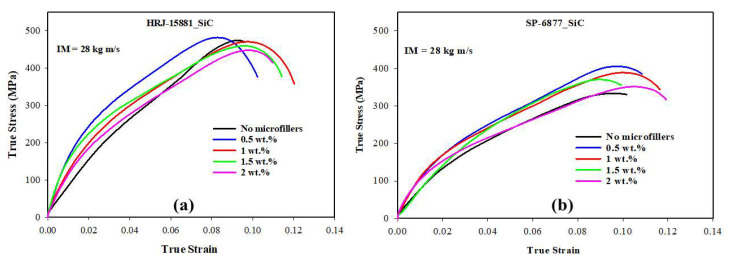
Typical true stress–true strain curves of SiC filler-modified CFRP composites (IM = 28 kg m/s): (**a**) HRJ-15881 and (**b**) SP-6877.

**Figure 19:**
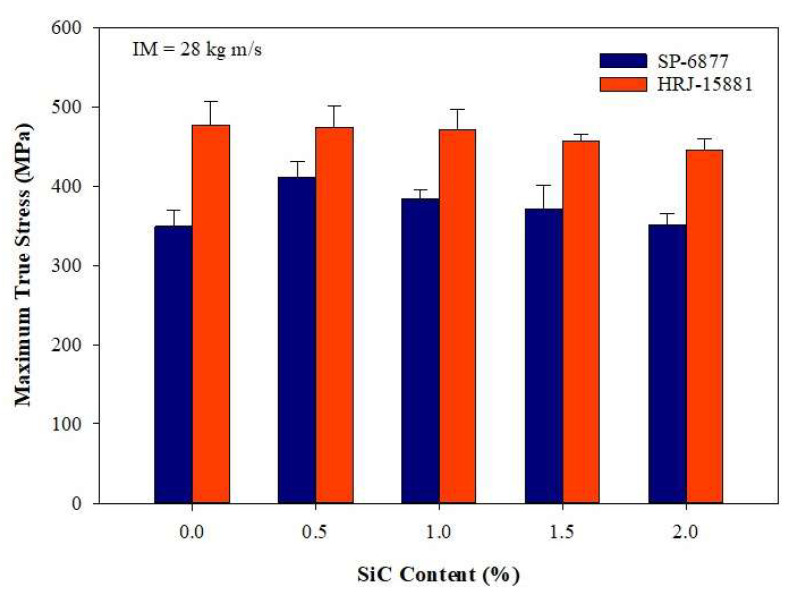
Comparison of maximum true stresses of SiC filler-modified CFRP composites based on the two resins (IM = 28 kg m/s). Error bars are based on standard deviation.

**Figure 20:**
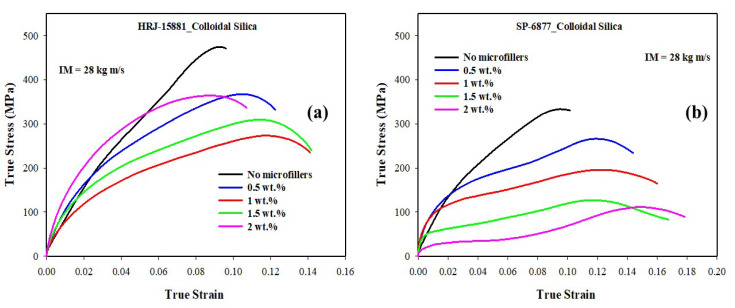
Typical true stress–true strain curves of CFRP composites modified with CS filler (IM = 28 kg m/s): (**a**) HRJ-15881 and (**b**) SP-6877.

**Figure 21:**
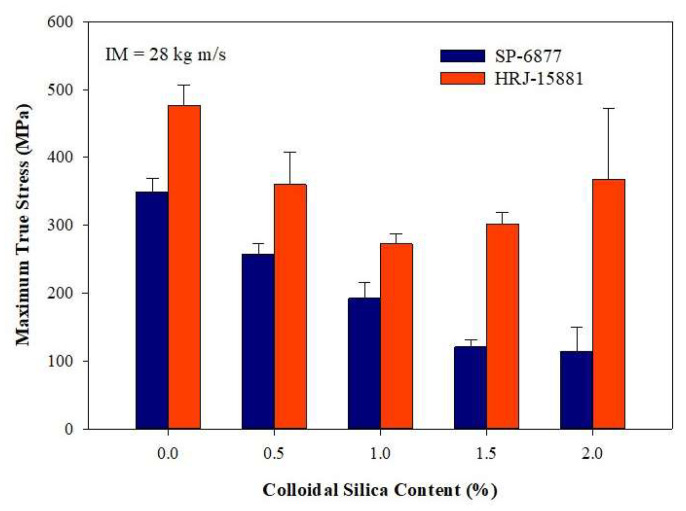
Comparison of maximum true stresses of CS filler-modified CFRP composites based on the two resins (IM = 28 kg m/s). Error bars are based on standard deviation.

Figure 17, Figure 18, Figure 19, Figure 20 and Figure 21 show the results of SHPB tests performed on specimens of CFRP composites at a momentum of 28 kg m/s. Figure 17 shows the true stress vs. true strain curves obtained for unmodified CFRP composites, while Figure 18 and Figure 19 show, respectively, the true stress–true strain curves and maximum true stresses obtained for SiC filler-modified composites. Graphs of true stress vs. true strain and maximum true stresses obtained for CS filler-modified composites are shown, respectively, in Figure 20 and Figure 21.

Figure 17 shows that the impact strength of unmodified CFRP composites based on HRJ-15881 matrix is higher than that based on SP-6877 matrix, which is consistent with the results of unmodified composites tested at 15 kg m/s (see Figure 12). Figure 18 and Figure 19 show that, within the range of filler content tested in this study, the impact strength of SiC filler-modified CFRP composites based on HRJ-15881 matrix is higher than that of those based on SP-6877 resin. A close examination of the true stress–true strain curves in Figure 18 shows that modification of SP-6877 resin with SiC filler improved the impact strength of the resulting composites above that of the unmodified composite at all SiC contents. In contrast, impact strength improvement is seen in HRJ-15881-based composites at only 0.5 wt.% SiC filler addition. Further addition of SiC filler beyond this level resulted in a decrease in impact strength of the resulting composites.

As reported previously for composites tested at 15 kg m/s, Figure 20 and Figure 21 show that modification of both resins with CS filler degraded the impact resistance of the resulting CFRP composites. Nevertheless, within the range of CS content studied, the impact strength of CFRP composites based on HRJ-15881 resin is higher those based on SP-6877 matrix at all CS contents. Furthermore, the impact strength of composites based on SP-6877 matrix generally decreased with increasing CS content. In contrast, the decrease in the impact strength of composites based on HRJ-15881 matrix did not follow a consistent trend. For all intents and purposes, the trend of the impact strength results obtained for composites tested at 15 kg m/s is similar to those tested at 28 kg m/s.

It is evident from the preceding results of SHPB tests, conducted at 15 kg m/s and 28 kg m/s, that the modification of HRJ-15881 and SP-6877 phenolic matrices with SiC filler enhanced the impact resistance of their respective CFRP composites. In contrast, modifying the resins with CS reduced the impact strength of the resulting composites. Broadly, the trends of impact strengths obtained in the present study are consistent with the previous work by Abulganiyu et al. [54] in which the flexural properties of CFRP composites improved with SiC filler addition, but deteriorated with CS addition due to the agglomeration of CS particles.

### 3.3. Microstructural Examination

Table 2 provides an overview of a visual examination of the failure conditions of specimens of CFRP composites subjected to SHPB testing at the two momentums. At 15 kg m/s, none of the specimens, irrespective of whether they were modified with SiC and CS microfillers or unmodified, fractured. Nevertheless, at 28 kg m/s, specimens of unmodified CFRP composite fractured. Since the fracture features observed for composites based on the two resins were similar, only CFRP composites based on HRJ-15881 matrix would be used as representatives to avoid duplication.

For SiC filler-modified CFRP composites tested at 28 kg m/s, only specimens modified with 0.5 and 1.0 wt.% SiC fractured. Again, as the failure features found in these two CFRP composites were similar, only the detailed microstructures of composites with 0.5 wt.% SiC will be presented. Specimens modified with 1.5 wt.% and 2.0 wt.% SiC filler suffered no fracture due to impact loading at 28 kg m/s. For CS-modified CFRP composites, no fracture was observed below 2.0 wt.% CS content. At 2.0 wt.% CS, specimens of composites based on both resins fractured. Again, failure features were similar for composites based on both resins and, as such, subsequent discussion will focus on HRJ-15881-based composites.

**Figure 22:**
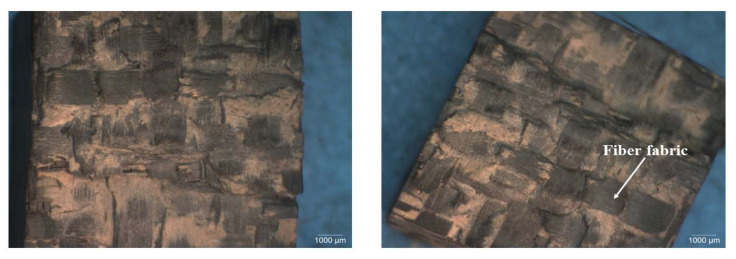
Typical optical macrographs of specimens of unmodified CFRP composites, based on HRJ-15881 resin, which were tested at 28 kg m/s.

**Figure 23:**
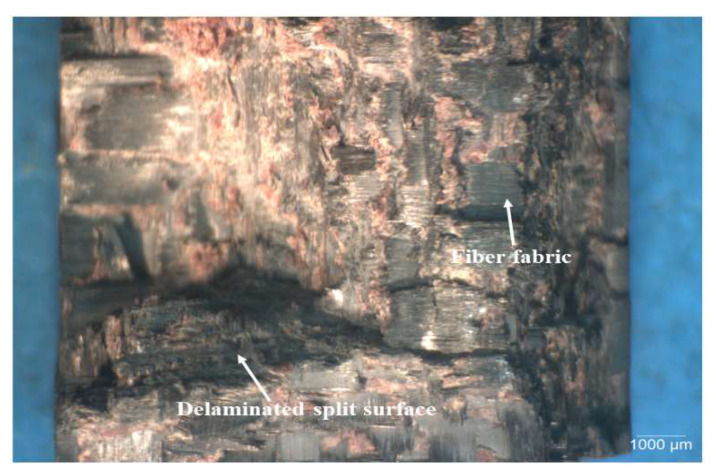
Typical optical macrograph of a specimen of HRJ-15881-based CFRP composite modified with 0.5 wt.% SiC tested at 28 kg m/s.

**Figure 24:**
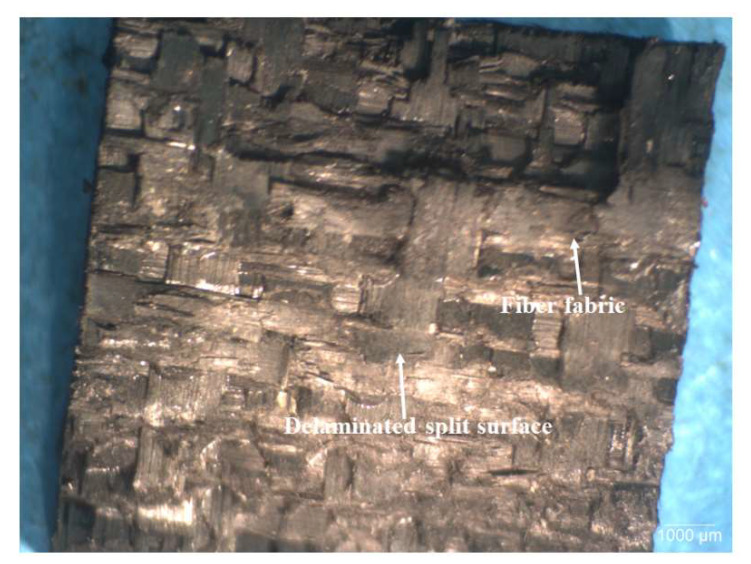
Typical optical macrograph a specimen of HRJ-15881-based CFRP composite modified with 2 wt.% CS tested at 28 kg m/s.

Figure 22, Figure 23 and Figure 24 show typical optical images of failed specimens of CFRP composites tested at 28 kg m/s. Figure 22 shows typical surfaces of failed specimens of unmodified CFRP composite, Figure 23 shows the same in the context of a CFRP composite modified with 0.5 wt.% SiC, while Figure 24 shows the same regarding a CFRP composite modified with 2.0 wt.% CS. The failure was typified by splitting the specimens into two, with the main failure mode being delamination. In addition, the failed specimens featured undulating, rough surfaces, which indicates that the matrix and fiber bundles ruptured during testing. This type of surface characteristic was more pronounced in SiC filler-modified CFRP composites than in those modified with CS filler. The surface morphology of composites modified with CS filler and those containing no fillers was similar.

**Figure 25:**
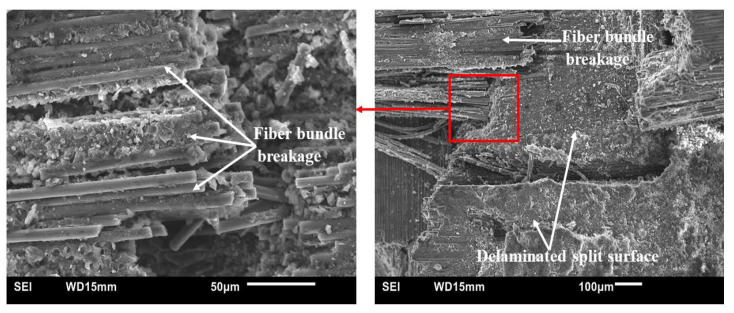
Typical SEM micrographs of unmodified HRJ-15881-based CFRP composites after SHPB testing at 28 kg m/s.

**Figure 26:**
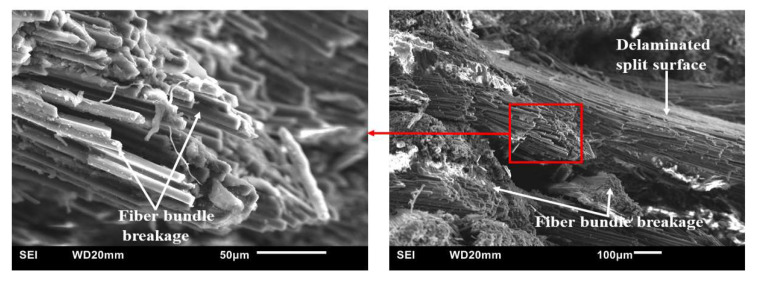
Typical SEM fractographs of HRJ-15881-based CFRP composite modified with 0.5 wt.% SiC after SHPB testing at 28 kg m/s.

**Figure 27:**
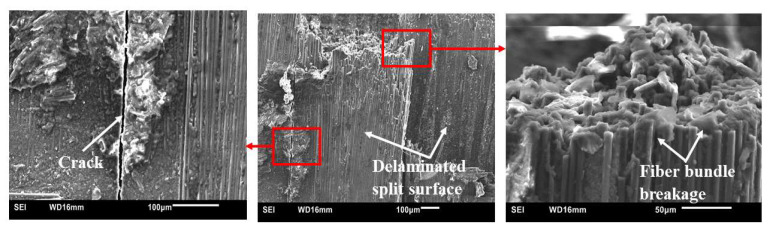
Typical SEM fractographs of HRJ-15881-based CFRP composites modified with 2 wt.% CS after SHPB testing at 28 kg m/s.

Figure 25, Figure 26 and Figure 27 present SEM micrographs of specimens of modified and unmodified CFRP composites that were tested 28 kg m/s. The main failure modes of the unmodified CFRP composites (Figure 25) were CF bundle rupture, specimen splitting, and delamination. Furthermore, in spite of the observed defects, it could be seen from Figure 25 that the CF fabric was impregnated by the phenolic resin quite well. The observed good adhesion between the matrix and CF could explain the reason why the fiber bundles remained intact despite rupture during impact testing.

Similarly, the failure mode of specimens of CFRP composites modified with 0.5 wt.% SiC (Figure 26) is also characterized by fiber bundle breakage and delamination. Nevertheless, a close comparison of Figure 25 and Figure 26 shows that fiber bundle breakage is more pronounced in the SiC filler-modified composites than in the unmodified composites. This may explain the basis for the presence of undulating, jagged ridged surfaces in the optical images of tested specimen shown previously in Figure 22, Figure 23 and Figure 24. Furthermore, Figure 26 shows there is fairly good bonding between the phenolic matrix and CF.

Figure 27 shows typical SEM micrographs of failed specimens of CFRP composites modified with 2 wt.% CS. As shown previously in Figure 25 and Figure 26, the failure mode is typified by CF bundle breakage and delamination. A matrix crack is shown in the image on the left. Moreover, the micrographs indicate there is good bonding between the matrix and CF, which could be attributed to the good impregnation of CF preforms by the resins.

## 4. Conclusions

The possibility of enhancing the resistance of CFRP composites to dynamic impact loading by modifying the matrix with SiC and CS microfillers was explored in this study. Plates of CFRP composites were produced using 2D woven CFs, microfillers, and two phenolic resins (SP-6877 and HRJ-15881). SHPB testing of specimens of the composites was conducted at two impact momentums, 15 kg m/s and 28 kg m/s. XRD diffractograms showed that the microfillers were intercalated in the phenolic resins. At 15 kg m/s, HRJ-15881-based CFRP composites modified with 1.5 wt.% SiC gave the highest impact strength. At 28 kg m/s, the impact strength of HRJ-15881-based CFRP composites was higher than that of the SP-6877-based CFRP composites at each level of SiC filler addition. In addition, in the range of SiC content used in this study (0.5–2.0 wt.%), the impact strength of SP-6877-based CFRP composites was better than that of its composite containing no SiC filler. In contrast, only the impact strength of HRJ-15881-based composite modified with 0.5 wt.% SiC was higher than its composite without SiC filler. Nevertheless, the modification of the two resins with CS degraded the impact strength of the resulting composites.

A microstructure examination showed that, at 15 kg m/s, no fracture occurred in the CFRP composites with and without microfillers. However, at 28 kg m/s, the CFRP composites failed by splitting, edge chip off, and crack extension through the matrix, which was more pronounced in the CFRP composites containing colloidal silica microfillers.

## Figures and Tables

**Table 1:** Compositions and properties of the two resole phenolic resins (HRJ-15881 and SP-6877).

Characteristics	HRJ-15881	SP-6877
Solids (%)	76.09	76.09
Phenol (%)	13.61	13.61
pH	8.1	8.1
Viscosity Brookfield (cP)	906	53.1
Gel Time (min.)	12.5	12.5
Formaldehyde (HCHO, (%))	0.5	1.3

**Table 2:** Visual inspection results obtained for CFRP composite specimens tested at two impact momentums.

Momentum	Filler Content	Did Fracture Occur?	
		HRJ-15881	SP-6877
15 kg m/s	None	No	No
28 kg m/s		Yes	Yes
15 kg m/s	0.5 wt.% SiC	No	No
	1.0 wt.% SiC	No	No
	1.5 wt.% SiC	No	No
	2.0 wt.% SiC	No	No
28 kg m/s	0.5 wt.% SiC	Yes	Yes
	1.0 wt.% SiC	Yes	Yes
	1.5 wt.% SiC	No	No
	2.0 wt.% SiC	No	No
15 kg m/s	0.5 wt.% CS	No	No
	1.0 wt.% CS	No	No
	1.5 wt.% CS	No	No
	2.0 wt.% CS	No	No
28 kg m/s	0.5 wt.% CS	No	No
	1.0 wt.% CS	No	No
	1.5 wt.% CS	No	No
	2.0 wt.% CS	Yes	Yes

## Data Availability

The data presented in this study are available on request from the corresponding author.
